# Rapid binding site analysis by means of structural interaction fingerprint patterns – an implication to GPCR-targeted CADD

**DOI:** 10.1186/1758-2946-3-S1-P42

**Published:** 2011-04-19

**Authors:** T Kosciolek, S Mordalski, AJ Bojarski

**Affiliations:** 1 Department of Medicinal Chemistry, Institute of Pharmacology, Polish Academy of Sciences, Smetna 12, 31-343 Krakow, Poland

## 

One of the most troublesome stages of Computer Aided Drug Design (CADD) process is analyzing huge amount of data provided by docking studies. Simple scoring functions alone can provide only shallow information about ligand-receptor interactions, since they do not distinguish neither residues nor single atoms. Very often a visual inspection is the only way to determine a binding mode. Here, we introduce an implementation of interaction profiles [1] based on Structural Interaction Fingerprints (SIFt) [2], which allow precise and rapid binding site description.

SIFts employed in our modelling agenda form a nine-digit binary pattern for each amino-acid in the receptor, covering all major types of interactions.

Computed over an ensemble of ligands and/or receptor conformations, averaged real number SIFt elegantly depicts overall preferences towards particular interactions. This enables construction of a compact scheme of crucial forces involved in ligand-receptor complex formation, which then facilitate design of a binding mode hypothesis and description of preferred ligand positions within the active site. An application is presented basing on a model of an allosteric domain of mGluR4 receptor [3] – a challenging computational task, due to untrivial evolutionary relationships for homology model construction purposes, and shortage of experimental data for hypotheses validation.
